# Physical mechanism of *δ*-*δ*′-*ε* phase stability in plutonium

**DOI:** 10.1038/s41598-017-06009-1

**Published:** 2017-07-17

**Authors:** Chun-Mei Li, Börje Johansson, Levente Vitos

**Affiliations:** 1 College of Physical Science and Technology, Shenyang Normal University, 110034 Shenyang, China; 2 Shenyang National Laboratory for Materials Science, Institute of Metal Research, Chinese Academy of Sciences, 72 Wenhua Road, 110016 Shenyang, China; 3 Department of Materials Science and Engineering, KTH - Royal Institute of Technology, 10044 Stockholm, Sweden; 4 Department of Physics and Astronomy, Division of Materials Theory, Uppsala University, Box 516, 75120 Uppsala, Sweden; 5 School of Physics and Optoelectronic Technology & College of Advanced Science and Technology Dalian University of Technology, 116024 Dalian, China; 6 Research Institute for Solid State Physics and Optics, Wigner Research Center for Physics, P.O. Box 49, HU-1525 Budapest, Hungary

## Abstract

Based on first-principle calculations, we have systematically explored the nature of the elastic stability and the *δ*-*δ*′-*ε* phase transitions in pure Pu at high temperature. It is found that, both the electron-phonon coupling and the spin fluctuation effects tend to decrease the tetragonal elastic constant (*C*′) of *δ*-Pu, accounting for its anomalous softening at high temperature. The lattice thermal expansion together with the electron-phonon coupling can stiffen *C*′ of *ε*-Pu, promoting its mechanical stability at high temperature. The *δ*-*ε* transition is calculated to take place around 750–800 K, and is dominated by the phonon vibration. The *δ*′ intermediate phase is realized around 750 K mainly because of the thermal spin fluctuation.

## Introduction

Plutonium is one of the most exotic elemental metals because of its condensed matter properties, metallurgy, and six allotropic phases^1–4^. Stable between 592 K and 724 K, the face-centered cubic (*δ*) phase has the lowest density and good ductility, and turns out to be technologically the most important phase, attracting a lot of research. It was reported to possess a negative thermal expansion coefficient (*α*), elastic anisotropy ($2{C}_{44}/({C}_{11}-{C}_{12})$) of about 7^1^, and an “abnormal” softening of the elastic modulus with temperature^5, 6^, which is coupled to the *δ*-*δ*′-*ε* transitions around 750 K. All these extraordinary thermodynamic properties make *δ*-Pu highly interesting, but the mechanisms behind its phase transitions are still not understood.

The anomalous thermophysical properties of *δ*-Pu are expected to be related to the itinerant-to-localized crossover of 5*f* electronic states^2, 7–12^. Within the generalized gradient approximation (GGA), the electronic total energy calculations have successfully reproduced the equilibrium volume (*V*) of *δ*-Pu^13^. Its large elastic anisotropy was evaluated to be about 8.3 by the GGA^13^ and 6.3 by the local density approximation (LDA) + U^14^ calculations. Nevertheless, these 0 K calculations generally gave inaccurate values for the elastic constants themselves and especially for $C^{\prime} =({C}_{11}-{C}_{12})/2$ ^13–15^, for which the estimated data (7.9 GPa^13^) is more than 60% larger than the experimental one (4.9 GPa^16^). A better description of *δ*-Pu requires that the electronic structure theories go beyond the ground state, by including the effects of temperature and electron-phonon coupling in the 5*f* band picture^17, 18^.

Similar to other metals and metallic alloys, the temperature-dependent lattice thermal expansion, magnetism, and phonon vibration may be considered as important factors^19, 20^. The thermal expansion effect on the bulk modulus (*B*) of *δ*-Pu has been theoretically examined^21^, whereas its influence on *C*′ of the *δ* and *ε* (body-centered cubic) phases were seldom investigated. Supported by the recent studies from both neutron-scattering experiments and phonon dispersions calculations^22–24^, *δ*-Pu is theoretically approximated with the paramagnetic (PM) state^25–27^, often described with the fully disordered local magnetic (DLM) model^28^. The dynamical fluctuations of the magnetization density (spin fluctuations) with temperature were supposed to induce strong magnetovolume and magnetoelastic couplings^29^, which subsequently influence the stability of the two phases and also the transition between them.

In this letter, we explore the elastic properties of the *δ* and *ε* phases and the *δ*-*δ*′-*ε* transitions of the PM state of Pu, taking all the temperature-dependent electronic and magnetic entropy, electron-phonon coupling, lattice thermal expansion, phonon vibration, and spin fluctuation effects into account, and try to uncover their physical mechanisms.

## Results and Discussion

**Figure 1:**
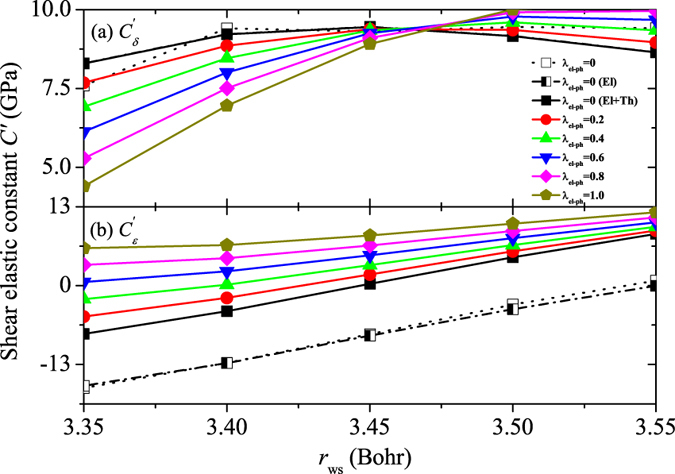
Static (0 K) tetragonal shear elastic constants *C*′ of *δ*- and *ε*-Pu as a function of Wigner-Seitz radius (denoted as “*λ*_el-ph_ = 0”). Shown are also results corresponding to 750 K including only the electronic entropy term (denoted as “*λ*_el-ph_ = 0 (El)”), both the electronic entropy and lattice thermal expansion terms (denoted as “*λ*_el-ph_ = 0 (El + Th)”), and the electronic entropy, lattice thermal expansion, and electron-phonon coupling three effects (denoted as “*λ*_el-ph_ = $0.2,0.4,\ldots ,1.0$”).

Figure 1 describes the calculated electronic entropy, lattice thermal expansion, and electron-phonon coupling effects on the *C*′ of *δ*- (${C}_{\delta }^{^{\prime} }$) and *ε*-Pu (${C}_{\varepsilon }^{^{\prime} }$). Here *T* is fixed to 750 K, i.e., close to the critical temperature where the *δ*-*δ*′-*ε* transitions occur^1^. We vary the Wigner-seitz radius (*r*_ws_) from 3.35 Bohr to 3.55 Bohr and the electron-phonon coupling coefficient (*λ*_el–ph_) from 0 to 1, with an interval of 0.05 Bohr and 0.2, respectively. At 750 K, the electronic entropy has practically no influence on ${C}_{\delta }^{^{\prime} }$ and ${C}_{\varepsilon }^{^{\prime} }$. The 0 K ${C}_{\delta }^{^{\prime} }$ changes non-monotonically with increasing *r*_ws_ (Fig. 1a), and in the studied *r*_ws_ range it changes only very little. This means that the lattice thermal expansion can change ${C}_{\delta }^{^{\prime} }$ by less than 2 GPa at 750 K. For the *ε* phase, the thermal expansion is positive. Shown in Fig. 1b, ${C}_{\varepsilon }^{^{\prime} }$ increases linearly with increasing *r*_ws_ at 0 K, thus the thermal expansion increases ${C}_{\varepsilon }^{^{\prime} }$. Actually, the lattice thermal expansion promotes the mechanical stability of the *ε* phase at high temperature.

Including the electron-phonon coupling at 750 K, ${C}_{\delta }^{^{\prime} }$ (Fig. 1a) increases above (decreases below) *r*_ws_ = 3.47 Bohr with increasing *λ*_el–ph_. For *λ*_el–ph_ = 1.0, the electron-phonon coupling changes the trend of ${C}_{\delta }^{^{\prime} }\sim {r}_{{\rm{ws}}}$ into a monotonically increasing ${C}_{\delta }^{^{\prime} }$ with *r*_ws_ at 750 K. At the experimental volume of the *δ* phase (around 3.43 Bohr^1^), the ${C}_{\delta }^{^{\prime} }$ value corresponding to *λ*_el–ph_ = 1.0 is much closer to the experimental data^16, 30^ than its static value. The ${C}_{\varepsilon }^{^{\prime} }$ (Fig. 1b) increases at each volume with *λ*_el–ph_. Like the lattice thermal expansion, the electron-phonon coupling tends to increase ${C}_{\varepsilon }^{^{\prime} }$, helping to realize the mechanical stability of this phase at high temperature.

**Figure 2:**
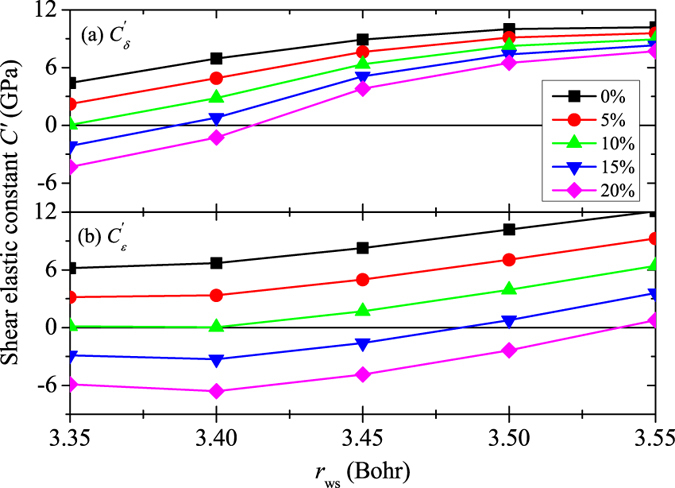
Tetragonal shear elastic constants *C*′ of *δ*- and *ε*-Pu as a function of Wigner-Seitz radius. Results correspond to 750 K, including the electronic entropy, lattice thermal expansion, electron-phonon coupling (*λ*_el-ph_ = 1.0), and spin fluctuation effects modelled by reducing *μ*_Pu_ by 0%, 5%, …, 20% relative to the static value.

Including all the above three effects evaluated with *λ*_el–ph_ = 1.0, we now investigate the impact of spin fluctuation on ${C}_{\delta }^{^{\prime} }$ and ${C}_{\varepsilon }^{^{\prime} }$ at 750 K, by performing calculations with the local magnetic moments of Pu (*μ*_Pu_) reduced by 0% to 20% relative to their equilibrium values. The physical picture behind this reduction is given by the longitudinal thermal spin fluctuations^31^, which is due to the particular energy versus magnetic moment curve of Pu stabilizing lower local magnetic moments at high temperature, as compared to the static DLM moment. As shown in Fig. 2, both ${C}_{\delta }^{^{\prime} }$ and ${C}_{\varepsilon }^{^{\prime} }$ become smaller with decreasing *μ*_Pu_. Therefore the spin fluctuation should present a significant contribution to the softening of the elastic moduli of *δ*- and *ε*-Pu with increasing *T* ^5, 6^. With *μ*_Pu_ reduced no more than 10%, ${C}_{\delta }^{^{\prime} }$ and ${C}_{\varepsilon }^{^{\prime} }$ remain positive within the considered volume range. With more than 10% reduction of *μ*_Pu_, they become negative, demonstrating the mechanical instability of the *δ* and *ε* phases at 750 K. Hence, around this temperature, *μ*_Pu_ may be no less than 90% of its static value for the two phases. The experimental ${C}_{\delta }^{^{\prime} }$ is available. According to Fig. 2a, with 10% reduced *μ*_Pu_, the evaluated ${C}_{\delta }^{^{\prime} }$ (4.9 GPa) at 750 K corresponding to the experimental volume (3.43 Bohr^1^) is in perfect agreement with the measured values (4.9 GPa^16^ and 4.8 GPa^30^). We notice that the ~0.5 *μ*_B_ reduction of the local magnetic moments obtained in the above semi-empirical estimation is surprisingly close to the predicted one based on ab initio spin fluctuation study^31^.

In what follows, we explore the *δ*-*δ*′-*ε* transitions by calculating the free energy (*F*) change with respect to *r*_ws_ and *c*/*a* of the body-centered-tetragonal Pu. Here, *c*/*a* = 1 represents the *ε* phase whereas *c*/*a* = 1.414 corresponds to the *δ* phase. The other values of *c*/*a* may correspond to the *δ*′ phase. First, the static electronic total energy (*E*_el_) term is found to prefer the *δ* phase with *c*/*a* = 1.414. The static electronic entropy (*TS*_el_) and magnetic entropy (*TS*_mag_) terms lower the free energy but, their effects are almost the same for the two structures. As a result, the *δ* phase is always stabilized whereas the *δ*′ and *ε* phases could not be obtained by considering only the above three terms into account.

**Figure 3:**
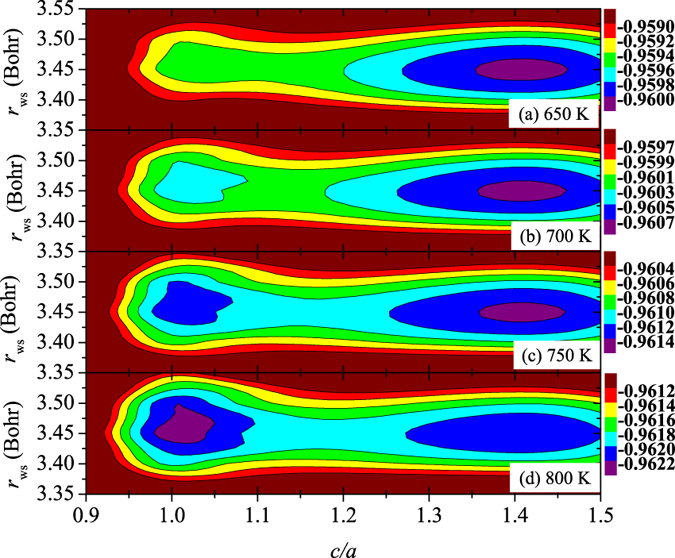
Free energy ($F={E}_{{\rm{el}}}-T{S}_{{\rm{el}}}+{F}_{{\rm{vib}}}-T{S}_{{\rm{mag}}}$, in unit of Ry) change with respect to the Wigner-Seitz radius (*r*_ws_) and *c*/*a* of Pu using the equilibrium *μ*_Pu_. Results are shown for 650 K (**a**), 700 K (**b**), 750 K (**c**), and 800 K (**d**), respectively. The two minima at *c*/*a* = 1.414 and *c*/*a* = 1 correspond to the *δ* and *ε* phases.

Adding the phonon vibrational free energy (*F*_vib_) in Fig. 3, it is still only the *δ* phase which is stable below 650 K. When *T* goes up to 700 K, a metastable phase appears around *c*/*a* = 1, which corresponds to the *ε* phase and with the relative free energy to the *δ* phase (${\rm{\Delta }}{F}_{\varepsilon -\delta }={F}_{\varepsilon }-{F}_{\delta }$, with *F*_*δ*_ and *F*_*ε*_ being *F* for the *δ* and *ε* phases, respectively) is about 0.35 mRy. With further increase of *T*, Δ*F*_*ε*−*δ*_ is gradually lowered to about 0.15 mRy at 750 K, and then to −0.12 mRy around 800 K. It indicates that with increasing *T*, the *ε* phase becomes more and more stable with respect to the *δ* phase. Around 800 K, the *ε* phase becomes lower in energy than the *δ* phase, and thus the *δ*-*ε* transition occurs. The *δ*′ intermediate phase, however, is not yet present.

**Figure 4:**
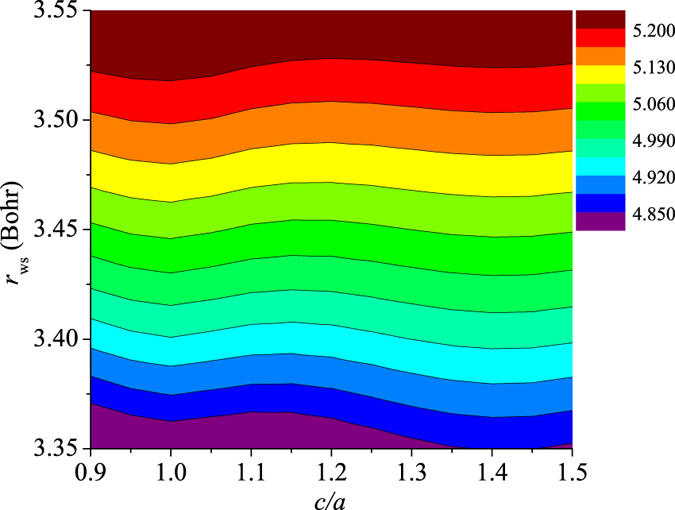
Equilibrium local magnetic moment of Pu (*μ*_Pu_, in unit of *μ*_B_) plotted as a function of the Wigner-Seitz radius (*r*_ws_) and *c*/*a*.

Shown in Fig. 4, the equilibrium (static) *μ*_Pu_ value depends on both *r*_ws_ and *c*/*a*. As it is generally expected, *μ*_Pu_ increases with *r*_ws_ at fixed *c*/*a*. On the other hand, corresponding to each *r*_ws_, *μ*_Pu_ non-monotonically changes with *c*/*a*, and the values for the *δ* (*c*/*a* = 1.414) and *ε* (*c*/*a* = 1) phases are larger than those of other (low symmetry) structures. Therefore, it is expected that the decrease of *μ*_Pu_ with temperature should have an influence on the structural stability of Pu.

**Figure 5:**
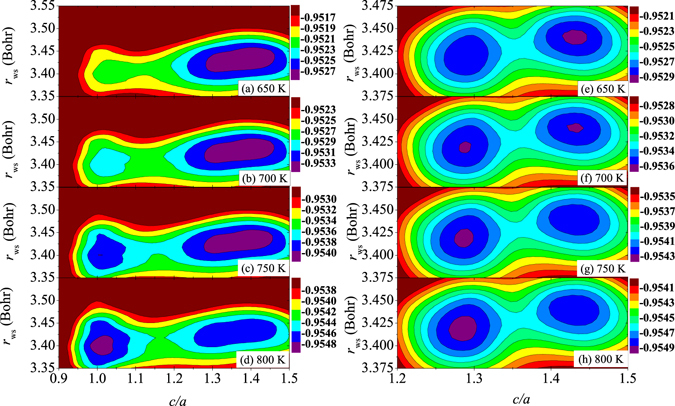
Free energy ($F={E}_{{\rm{el}}}-T{S}_{{\rm{el}}}+{F}_{{\rm{vib}}}+{E}_{{\rm{mag}}}-T{S}_{{\rm{mag}}}$, in unit of Ry) change with respect to the Wigner-Seitz radius (*r*_ws_) and *c*/*a* of Pu using 10% reduced *μ*_Pu_. Results are shown for 650 K (**a**,**e**), 700 K (**b**,**f**), 750 K (**c**,**g**), and 800 K (**d**,**h**), respectively. The three minima correspond to the *δ*, *δ*′, and *ε* phases in the left panel, whereas in the right panel, the two minima indicate the *δ* and *δ*′ phases.

Next, we take the spin fluctuation into account by calculating *F* with 10% reduction of the equilibrium *μ*_Pu_, and draw *F* as a function of *r*_ws_ and *c*/*a*. The results are shown in Fig. 5. In the left panel of the figure, with increasing temperature, the calculated Δ*F*_*ε*−*δ*_ goes down from 0.46 mRy at 650 K to 0.28 mRy at 700 K, to 0.02 mRy at 750 K, and then to −0.18 mRy at 800 K. Therefore, similar to Fig. 3, the *δ*-*ε* transition is again obtained around 750–800 K. Hence, by reducing *μ*_Pu_ by 10%, the relative stability between the two cubic phases does not change significantly.

Very interestingly, with reduced local magnetic moment, another phase appears around *c*/*a* = 1.28. We propose that this metastable phase corresponds to the *δ*′ phase. In order to highlight the stability of the *δ*′ phase relative to the *δ* one, the energy map corresponding to these two structures is plotted in details in the right panel of Fig. 5. The two minima located around *c*/*a* = 1.414 and *c*/*a* = 1.28 correspond to the *δ* and *δ*′ phases. With the increase of *T*, the free energy of the *δ*′ phase relative to that of the *δ* one (Δ*F*_*δ*′−*δ*_ = *F*_*δ*′_ − *F*_*δ*_, with *F*_*δ*′_ being *F* for the *δ*′ phase) decreases from 0.11 mRy at 650 K to 0 mRy at 700 K, to −0.04 mRy at 750 K, and then to −0.08 mRy at 800 K. This means that the *δ*′ phase is stabilized above 650 K. With increasing *T* above 750 K, the *δ*′ phase becomes more stable than the *δ* phase, indicating the *δ*-*δ*′ transition. In comparison the left and right panel of Fig. 5, the *δ*′ phase is stabilized only in a short temperature range around 750 K, since at 800 K, the *ε* phase is stabilized instead with about −0.10 mRy lower energy than that of the *δ*′ phase.

The volumes of the *δ*, *δ*′, and *ε* phases evaluated with 10% reduced *μ*_Pu_ are about 25.2 Å^3^, 24.8 Å^3^, and 24.4 Å^3^, respectively, all in good agreement with the experimental data (25.2 Å^3^, 25.1 Å^3^, and 24.4 Å^3^, respectively^1^). The obtained *c*/*a* value (1.28) of the *δ*′ phase in Fig. 5 is also in line with the experimental data (1.34^1^). We should point out that in Fig. 3, where the *F* is calculated with the equilibrium *μ*_Pu_, the volumes of the *δ* and *ε* phases are about 25.5 Å^3^ and 25.8 Å^3^, respectively, which are larger than the experimental ones^1, 16, 30^. Hence, the spin fluctuation tends to favor the stability of the *δ*′ phase relative to the *δ* phase, and also contributes to the reduction of *V* during the *δ*-*δ*′-*ε* transitions of Pu.

**Figure 6:**
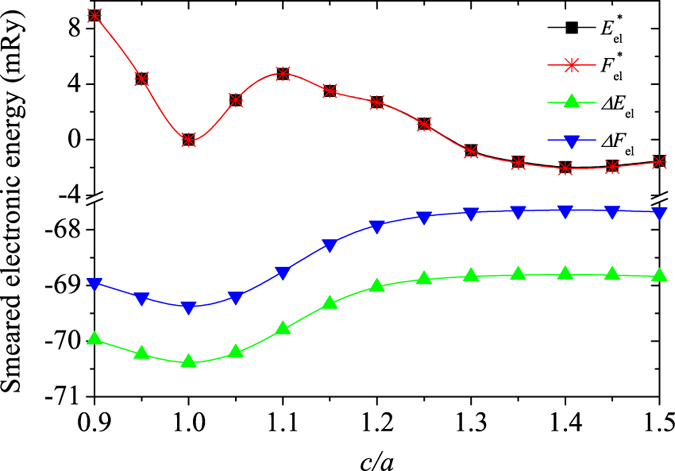
Smeared electronic energies (${E}_{{\rm{el}}}^{\ast }$ and ${F}_{{\rm{el}}}^{\ast }={E}_{{\rm{el}}}^{\ast }-T{S}_{{\rm{el}}}^{\ast }$) and their differences relative to the static values (${\rm{\Delta }}{E}_{{\rm{el}}}={E}_{{\rm{el}}}^{\ast }-{E}_{{\rm{el}}}$ and ${\rm{\Delta }}{F}_{{\rm{el}}}={F}_{{\rm{el}}}^{\ast }-{F}_{{\rm{el}}}$) with respect to *c*/*a* of Pu. The *ε* phase (*c*/*a* = 1) is used as reference for both the ${E}_{{\rm{el}}}^{\ast }$ and ${F}_{{\rm{el}}}^{\ast }$ curves.

Using the equilibrium volume of the *δ* phase, we calculate the 0 K density of states (DOS) change with respect to *c*/*a*, and then evaluate the smeared DOS and the corresponding energies (${E}_{{\rm{el}}}^{\ast }$ and ${F}_{{\rm{el}}}^{\ast }={E}_{{\rm{el}}}^{\ast }-T{S}_{{\rm{el}}}^{\ast }$, with ${E}_{{\rm{el}}}^{\ast }$, ${F}_{{\rm{el}}}^{\ast }$, and $T{S}_{{\rm{el}}}^{\ast }$ being the smeared electronic total energy, smeared free energy, and smeared electronic entropy, respectively) with *λ*_el–ph_ = 1.0 at 750 K. In Fig. 6, the obtained *c*/*a*-dependence of ${E}_{{\rm{el}}}^{\ast }$, ${F}_{{\rm{el}}}^{\ast }$, and their differences relative to the static values (${\rm{\Delta }}{E}_{{\rm{el}}}={E}_{{\rm{el}}}^{\ast }-{E}_{{\rm{el}}}$ and ${\rm{\Delta }}{F}_{{\rm{el}}}={F}_{{\rm{el}}}^{\ast }-{F}_{{\rm{el}}}$, with ${F}_{{\rm{el}}}={E}_{{\rm{el}}}-T{S}_{{\rm{el}}}$ being the static electronic free energy) are compared. Here, the *ε* phase (*c*/*a* = 1) is used as reference for both the ${E}_{{\rm{el}}}^{\ast }$ and ${F}_{{\rm{el}}}^{\ast }$ evaluations. At each *c*/*a* value, ${E}_{{\rm{el}}}^{\ast }$ and ${F}_{{\rm{el}}}^{\ast }$ have almost the same values whereas Δ*F*_el_ is larger than Δ*E*_el_, indicating that the electron-phonon coupling tends to evenly increase the -*TS*_el_ of each structure, and therefore still does not really influence the relative stability of the three phases. Increasing *c*/*a* from 0.9 to 1.5, both ${E}_{{\rm{el}}}^{\ast }$ and ${F}_{{\rm{el}}}^{\ast }$ show two minima. One corresponds to the *δ* phase (*c*/*a* = 1.414) whereas the other one means the *ε* phase. Therefore, considering the electron-phonon coupling, both the *ε* and *δ* phases are stable, but the latter is lower in energy than the former one (at 750 K). The *δ*′ phase, nevertheless, still can not be realized by this term at 750 K. As seen from the curve of ${\rm{\Delta }}{E}_{{\rm{el}}}\sim c/a$, Δ*E*_el_ has its maximum value (about −68.82 mRy) for *c*/*a* ~ 1.30–1.45, whereas its minimum is around *c*/*a* = 1 (about −70.38 mRy). This confirms that at high temperature, the phonon smearing indeed lowers *E*_el_ of the *ε* phase relative to that of the *δ* or *δ*′ ($c/a\approx 1.30$) phase, promoting the stabilization of the *ε* phase relative to the other two phases. Adding this effect to *F*, the critical temperature of *δ*-*ε* or *δ*′-*ε* transition derived above could be a little bit further lowered, and then they would be close to the experimental data (about 753 K^1^). Therefore, the phonon vibration, spin fluctuation, and electron-phonon coupling effects all contribute to the *δ*-*δ*′-*ε* transitions of Pu at high temperature.

## Conclusion

In summary, using first-principles theory in combination with physically sound approximations, we have systematically explored the nature of the elastic stability of *δ*- and *ε*-Pu and the phase transitions of *δ*-*δ*′-*ε* at high temperature. It is found that, the temperature effects show great influence on both ${C}_{\delta }^{^{\prime} }$ and ${C}_{\varepsilon }^{^{\prime} }$. The electron-phonon coupling and the reduction of *μ*_Pu_ with temperature tend to reduce ${C}_{\delta }^{^{\prime} }$, accounting for its anomalous softening at high temperature. The lattice thermal expansion and the electron-phonon coupling stiffen ${C}_{\varepsilon }^{^{\prime} }$, together giving rise to the mechanical stability of the phase at high temperature. The transitions of *δ*-*δ*′-*ε* are controlled mainly by the phonon vibration, spin fluctuation, and electron-phonon coupling. The transition of *δ*-*ε* is observed around 750 K–800 K, and is dominated by the phonon vibration. The *δ*′ intermediate phase could be realized around 750 K because of the thermal spin fluctuation. The electron-phonon coupling further improves the stability of the *ε* phase relative to the other two phases. The present insight provides a good understanding of the nature of the elastic stability and the *δ*-*δ*′-*ε* phase transitions of Pu at high temperature, and gives a solid ground for further advanced theoretical investigations of the subject matter.

## Methods

The employed first-principle solver is the exact muffin-tin orbitals (EMTO) method in combination with the coherent potential approximation (CPA)^32–37^. The EMTO-CPA is one of the few possible approaches to deal with magnetic disorder at first-principle level. In the present self-consistent calculations, the exchange correlation is chosen to be GGA as described by Perdew, Burke, and Ernzerhof (PBE)^38^. The EMTO basis set includes *s*, *p*, *d*, and *f* components, and the scalar-relativistic and soft-core approximations are adopted. The Green’s function is calculated for 32 complex energy points on a semicircular contour. For the slope matrix, two-center Taylor expansion is used, and the number of orbitals for charge density is truncated at 8. The Brillouin zone is sampled by a 13 × 13 × 13 uniform *k*-point mesh.

The equation of state at 0 K is determined by fitting the calculated total energies versus volume to a Morse function^39^. The 0 K elastic constants *C*′ of the *δ* and *ε* phases, characterizing the softness of the cubic lattices against tetragonal deformation, are evaluated with the methods presented previously^40^, and their temperature dependence including electronic entropy, lattice thermal expansion, electron-phonon coupling, and magnetism are considered. The self-consistent calculations performed at each volume for different temperatures in the Fermi-Dirac distribution give the electronic entropy effect on *C*′. The variation of *C*′ due to the thermal expansion is calculated as ${\tfrac{dC^{\prime} }{d{r}_{{\rm{ws}}}}|}_{T=0}{r}_{0}\alpha T$, where *r*_0_ is the equilibrium Wigner-Seitz radius at 0 K. For the thermal expansion coefficient *α*, we choose the experimental values of the *δ*, *δ*′, and *ε* phases^1^, −9.0 × 10^−6^ K^−1^, −66.0 × 10^−6^ K^−1^, and 36.5 × 10^−6^ K^−1^, respectively. The phonon-smearing effect on *C*′ is obtained as the difference between the second-order strain derivatives of the electronic free energies *F*_el_, calculated using the smeared density of states and the bare density of states, respectively, using equation (2) in our previous paper^19^. In real metals at elevated temperatures, the electrons experience a smeared density of state (${N}^{\ast }(E)$) as a result of phonon-limited lifetime. This effect can be formulated as Lorentz-type smearing of the electronic density of state^41^

1 $${N}^{\ast }(E)={\int }_{\infty }^{\infty }\,N(\varepsilon )\frac{({\rm{\Gamma }}/\pi )}{{(E-\varepsilon )}^{2}+{{\rm{\Gamma }}}^{2}}d\varepsilon ,$$

where Γ = *πλ*_el-ph_ *k*_*B*_ *T* is inverse proportional with the electron lifetime, with *k*_B_ being the Boltzmann constant. For most of the metals electron-phonon parameter *λ*_el-ph_ is of order of unity^41^. In the present study, *λ*_el-ph_ is set as 0, 0.2, …, and 1, respectively, to explore the phonon-smearing effect on *C*′, whereas to investigate this effect on the free energy, *λ*_el-ph_ = 1 is adopted.

The thermal spin fluctuations influence the mean local magnetic moments and thus the free energy and ultimately also the elastic constants of Pu. In a recent density functional theory study^31^, the thermal spin fluctuation effect for *δ*-Pu was found to decrease the value of the mean magnetic moment by ~0.5 *μ*_B_ as the temperature increases from 0 K to 700 K. The reduction of the local magnetic moment was found to be critical to explain the observed softening of the bulk modulus with increasing temperature. Based on these reported data, the spin fluctuation effect is here considered by calculating *C*′ with *μ*_Pu_ reduced by 0%, 5%, …, 20% relative to the equilibrium (static) value. To further investigate the spin fluctuation effect on the free energy, 10% reduce of the equilibrium *μ*_Pu_ value is used, which is similar to the ab initio reduction predicted in the above study^31^.

The Helmholtz free energy (*F*) is decomposed as

2 $$F={E}_{{\rm{el}}}-T{S}_{{\rm{el}}}+{F}_{{\rm{vib}}}+{E}_{{\rm{mag}}}-T{S}_{{\rm{mag}}}.$$

Here, the *E*_el_ and *S*_el_ are calculated directly with the EMTO method. Since the *δ*-*δ*′-*ε* transitions of Pu occur above 650 K, the phonon vibrational free energy, *F*_vib_, is approximated by its high temperature expansion, ${F}_{{\rm{vib}}}\approx 3{k}_{{\rm{B}}}T\frac{{\rm{\Theta }}-{{\rm{\Theta }}}_{0}}{{\rm{\Theta }}}$ ^41^, with ${\rm{\Theta }}$ and ${{\rm{\Theta }}}_{0}$ being the Debye temperatures corresponding to a volume of a tetragonal structure (specified in terms of Wigner-Seitz radius *r*_ws_ and *c*/*a*) and to the equilibrium one of the *δ* phase, respectively. According to the simplest approximation which has been applied to several metals^42^, the Debye temperature is proportional to $\sqrt{{r}_{{\rm{ws}}}B}$. Its temperature dependence is then easily evaluated by including the lattice thermal expansion and magnetic effects on the bulk modulus *B*, which are calculated with the same methods adopted above for *C*′. The magnetic energy, *E*_mag_, is calculated as ${E}_{{\rm{mag}}}={E}_{{\rm{el}}}^{{\rm{fix}}}-{E}_{{\rm{el}}}$, where ${E}_{{\rm{el}}}^{{\rm{fix}}}$ and *E*_el_ are the static electronic total energies corresponding to the fixed and equilibrium *μ*_Pu_ values, respectively. The magnetic entropy, *S*_mag_, is evaluated at each *μ*_Pu_ using the mean-field expression ${S}_{{\rm{mag}}}={k}_{{\rm{B}}}\,\mathrm{ln}\,({\mu }_{{\rm{Pu}}}+1)$ ^43^.
